# Coronary Sinus Phlebography in Cardiac Resynchronization Therapy Patients: Identifying and Solving Demanding Cases

**DOI:** 10.19102/icrm.2020.110703

**Published:** 2020-07-15

**Authors:** Lenine Angelo Alves Silva, Enoch Brandão de Souza Meira, Jefferson Curimbaba, João A. Pimenta

**Affiliations:** ^1^Division of Cardiovascular System, Hospital Universitário Lauro Wanderley, Universidade Federal da Paraíba, João Pessoa, Brazil; ^2^Division of Cardiovascular Surgery, IAMSPE—Hospital do Servidor Público Estadual, São Paulo, Brazil; ^3^Division of Cardiology, IAMSPE—Hospital do Servidor Público Estadual, São Paulo, Brazil

**Keywords:** Cardiac resynchronization therapy, coronary sinus access, coronary sinus phlebography, left ventricular lead placement

## Abstract

Cardiac resynchronization therapy (CRT) improves symptoms and reduces morbidity and mortality in select heart failure patients but remains challenging to deploy widely because of difficult or unsuccessful coronary sinus (CS) access in up to 10% to 15% of patients. This report describes the radiological and anatomical aspects for improving CS catheterization and left ventricular (LV) lead positioning, focusing on the radioscopic and anatomical aspects, based on phlebography, to identify demanding cases in patients with dilated cardiomyopathy referred for CRT implantation. Anatomical and radiological aspects were explored in the anteroposterior, 30° left anterior oblique, and 30° right anterior oblique (RAO) views. In total, 117 phlebographies were performed in 39 consecutive procedures (one reintervention). Access to the CS was successful 37 times (94.9%). The most difficult cases were complicated by issues related to the altered spatial orientation of the CS ostium toward the tricuspid annular plane (TAP), which was best perceived in the 30° RAO projection and occurred in 37% of patients. One of two catheterization failures that occurred was caused by anomalous coronary venous drainage into the left atrium. Final LV lead positioning was successful in 36 (92.3%) of 39 procedures. More severe heart failure and worse LV ejection fraction did not translate into greater difficulty in LV lead implantation. As such, understanding anatomical and radiological relationships is the key to successful LV lead positioning. RAO projection can be particularly useful in the assessment of demanding CRT implant cases, especially when the CS ostium pointed to the TAP.

## Introduction

Cardiac resynchronization therapy (CRT) improves symptoms and reduces morbidity and mortality in symptomatic heart failure patients with left bundle branch block and reduced ejection fraction.^1–8^ A fully transvenous CRT implant is preferable to using the combined transvenous and epicardial approach but it can be difficult to implement or unsuccessful in up to 10% of patients eligible for CRT,^9^ which has led to a search for alternatives to traditional resynchronization, such as His-bundle pacing.^10^ The major challenge inherent with the transvenous technique is the achievement of stable coronary sinus (CS) catheterization necessary to introduce the left ventricular (LV) lead and advance it into the coronary venous system.^11^ Difficulties usually arise from the anatomy of CS, spatial orientation of its ostium, and anatomical deformations attributable to cardiac dilatation or embryological variation. Obstructions in veins such as valves and kinking are also common.^12–14^ In addition to the operator’s experience, several techniques, including phlebography, coronary angiography, intracavitary electrogram, and the application of auxiliary catheters, have been described to facilitate CS catheterization.^15–19^ Despite being well-established as a treatment modality, the use of CRT remains challenging, mainly because of the nonresponder rate, and the risks of procedural failures reach 13% in most published trials.^15,20^ The search for alternative tools and techniques continues to reduce the complexity of CRT implantation and decrease the rate of LV lead placement failure. Despite the advances made, however, the technique for implantation is still dependent on radioscopy. In this context, the anatomical knowledge and its relationship with radiology are essential to achieving a higher success rate.

This report emphasizes the importance of phlebography in anteroposterior (AP), left anterior oblique (LAO), and right anterior oblique (RAO) projections in CRT. It also tries to identify difficult cases and search for a landmark to improve CS catheterization and LV lead placement by using simple anatomical and radiological aspects in patients with a classical indication for CRT. Finally, it reviews the concepts related to the applied radiological anatomy of the cardiovascular system, particularly in patients with dilated cardiomyopathy.

## Methods

### Patients

Patients with dilated cardiomyopathy who were candidates for CRT in a single-center experience were enrolled and included in this prospective study. The same operator performed all the procedures. To be a candidate for CRT, patients had to have dilated heart failure despite optimized medical therapy, New York Heart Association (NYHA) functional class III or IV symptoms, left bundle branch block with QRS duration of greater than 150 ms, an LV ejection fraction of less than 40%, and a life expectancy of not shorter than one year. This study was approved by an investigational review board and patient consent was gathered via a consent form.

### Implantation procedure

Implantations were performed in the catheterization laboratory. Patients were placed under local anesthesia and sedation when necessary. Prophylactic antibiotics were administered intravenously in all patients. The radiological anatomy of the CS was studied in AP, 30° LAO, and 30° RAO projections. Electrodes for electrocardiographic monitoring were attached to the patient’s back to avoid interference with the image of the cardiac silhouette.

**Figure 1:**
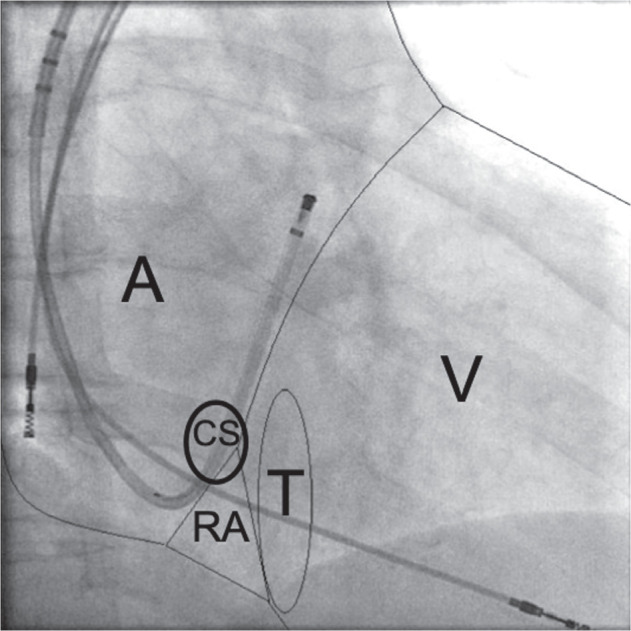
RAO projection at 30°, showing an inserted right atrial lead, a fixed RV lead, a deflectable catheter placed in the CS, and a schematic presentation of the anatomical–radiological cardiac silhouette. A: atrium; RA: radiolucent area; CS: coronary sinus; T: tricuspid annular plane; V: ventricle.

**Figure 2:**
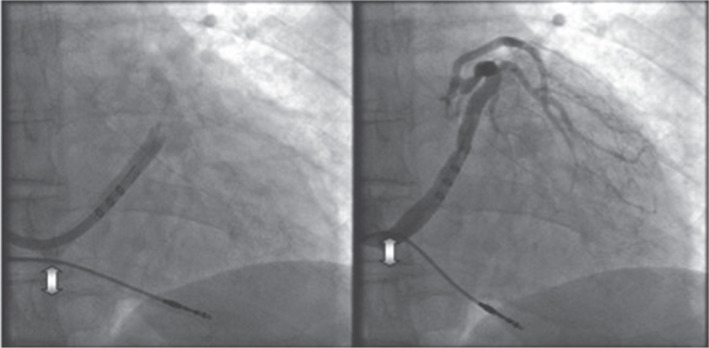
RAO projection at 30° during CS phlebography in the diastole (left) and at the end of systole (right). Arrows indicate RV lead motion at the site of the TAP.

The left cephalic vein was a preferred route for active fixation of the right atrial and right ventricular (RV) leads. An inserted atrial lead was not fixed before successful LV lead positioning, whereas an RV lead was immediately attached at the RV apex or midseptum, with the lead body left “to rest” at the base of the right atrium **(Figure 1)**. In this way, the systolic motion of the RV lead without the guidewire, seen in the 30° RAO projection, identified the tricuspid annular plane (TAP) **(Figure 2)**.

**Figure 3:**
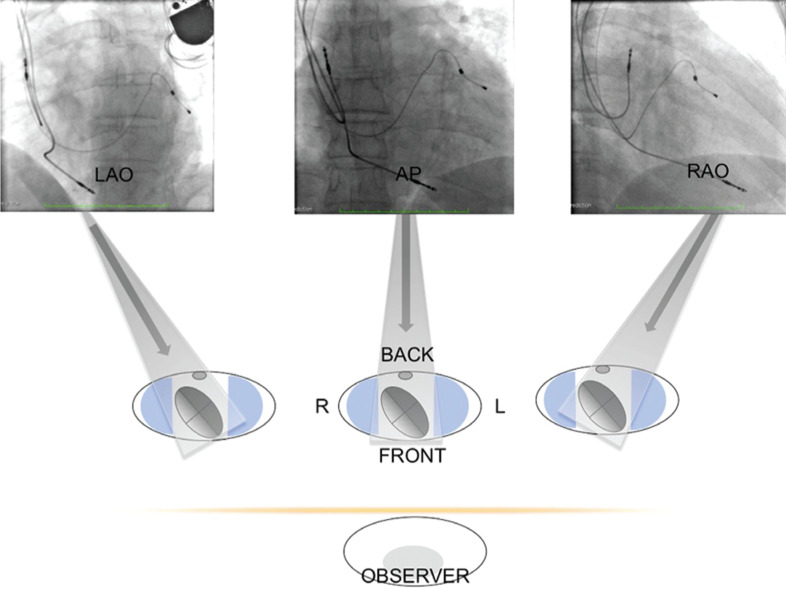
Spatial orientation of the cardiac chambers in the 30° LAO, AP, and 30° RAO projections. Observe the layout of the biventricular system. L: left; R: right.

For LV lead positioning, passive “over-the-wire” leads were used. Left subclavian or left axillary vein puncture was preferred to afford sufficient area for manipulation of the sheath and catheters for CS approach. After the puncture, a guiding sheath was positioned at the TAP. A guidewire, a preformed lead, or a deflectable catheter (preferred option) was introduced through the sheath to explore and cannulate the CS. CS phlebography was always performed in the three projections. The radiolucent area seen in the 30° RAO projection, corresponding to the atrioventricular groove, was used thereafter as a reference above, where we expected to find the CS on the left side and the TAP on the right side **(Figure 1)**.^21^ **Figure 3** shows the implanted leads in all three projections and clockwise and counterclockwise movements at the top and bottom of the radiolucent area facilitated CS catheterization. The position of the LV lead at the CS was confirmed in the 30° LAO view; in this context, the lead should point to and cross the line reference of the spinal column from right to left. Collected images were compared with previous CS phlebography records.

After cannulating the CS, the LV lead was advanced, preferably into the left marginal, the left posterior, or a middle cardiac vein, avoiding the great cardiac vein. The atrial lead was fixed before removing the LV guiding sheath to avoid displacement of the LV lead. The ideal LV site should demonstrate a low and stable LV pacing threshold (≤ 1.5 V at 0.40 ms) and no extracardiac stimulation at the high output (≥ 4.5 V at 0.50 ms). This triad, associated with the removal of the lead delivery system without lead displacement, guaranteed the success of the procedure. **Figure 3** shows the implanted leads in all three projections.

### Definition of variables

We analyzed the success rates and time intervals. CS catheterization was regarded as stable if there was secure positioning of the sheath and subsequent placement of the LV lead or the balloon catheter to perform phlebography. We defined “time from puncture to stable catheterization” as the time interval from central venous access to stable catheterization of the CS. Catheterization failure was defined as an inability to achieve stable catheterization within 120 minutes after puncture.

Likewise, “time from stable catheterization to final LV lead position” was defined as the time interval from stable CS catheterization to successful final LV lead positioning, with all tests done and the sheath removed. The sum of this time and the time from puncture to stable catheterization was defined as the “time from puncture to final LV lead position.” The reasons for failed LV lead positioning after successful CS catheterization included the inability to achieve stable LV lead position due to an anatomical barrier, high LV pacing threshold, and/or nonpreventable phrenic nerve stimulation. After each procedure, a questionnaire was applied to evaluate the perception of the level of difficulty related to CS catheterization and LV lead positioning.

### Statistical analysis

Data were tabulated in Excel 2011 for Mac® (Microsoft Corp., Redmond, WA, USA) and analyzed with the Statistical Package for the Social Sciences version 20.0 for Windows® (IBM Corp., Armonk, NY, USA). Continuous variables are reported as means ± standard deviations (minimum–maximum) and medians (interquartile ranges). Categorical data are reported as absolute and relative frequencies. All data were compared by sex. The time intervals defined in the previous section were additionally compared by LV ejection fraction (with the mean LV ejection fraction acting as the cutoff value), NYHA class, and spatial orientation of the CS ostium. Continuous data were non-normally distributed (assessed by the Kolmogorov–Smirnov–Lilliefors test) and were, hence, compared with the Mann–Whitney U test. Categorical data were compared using the exact Pearson’s chi-squared test. A two-sided p-value of less than 0.05 was considered to be statistically significant.

## Results

We performed 117 phlebographies in 38 consecutive patients (one reintervention) referred for CRT. Male sex slightly prevailed (55%) and male patients showed significantly worse ejection fraction (28% ± 6% versus 34% ± 3%; p = 0.008) and NYHA class (3.6 ± 0.4 versus 3.2 ± 0.3; p = 0.008) profiles than female patients despite nonsignificant younger ages in the former. The major causes of dilated cardiomyopathy were ischemia and valvular disease in males and hypertension and Chagas disease in females **(Table 1)**. About three-quarters of patients received CRT pacing systems (73.7%) and one-quarter received CRT defibrillation systems (26.3%) for secondary prevention.

All patients underwent one implantation procedure, except for one individual who experienced late LV lead dislodgment and required reintervention. Thus, the total number of procedures was 39, with 117 phlebographies. Serious complications included one case of CS perforation without hemodynamic consequences (diagnosed after phlebography) and one case of pneumothorax requiring surgical drainage. There were no deaths. As such, our complication rate of 5% fell within the range reported in the literature, where overall perioperative complication rates vary from 4% in more recent trials to as high as 28% in earlier CRT trials.^9^

The attempt made to access the CS was successful 37 times (success rate: 94.9%). One catheterization failure was caused by anomalous CS drainage into the left atrium as was confirmed by coronary angiography during the venous phase. The other catheterization failure occurred in the patient who required two procedures. In this patient, the LV lead was successfully positioned during the initial implantation but, when the LV lead dislodgment necessitated reintervention four months later, it was no longer possible to advance the LV lead into the cardiac veins. The initial implantation was classified as successful but the reintervention was deemed unsuccessful.

Final LV lead positioning succeeded in 36 (92.3%) of 39 procedures. The reasons for failures were catheterization problems, as mentioned above (n = 2), and unfavorable anatomy rendering phrenic nerve stimulation nonpreventable (n = 1). There were no significant differences between males and females concerning the success of stable CS catheterization or final LV lead position **(Table 1)**. Patients with unsuccessful transvenous implantations were referred for epicardial LV lead placement.

The mean time from puncture to stable CS catheterization was 17 minutes ± 22 minutes, ranging widely from two minutes to 98 minutes. The subsequent time from stable CS catheterization to the final LV lead position was 30 minutes ± 22 minutes (range: 10–107 minutes). The total time was 46 minutes ± 29 minutes (range: 13–121 minutes). Although there were large interpatient differences during all three time intervals, subdivisions according to sex **(Table 1)** and LV ejection fraction **(Table 2)** did not result in significant differences between the groups. The same was true after subdivision according to NYHA class **(Table 3)**. The more severe heart failure and worse LV ejection fraction observed in male patients, thus, did not translate into greater difficulty faced during LV lead implantation.

Regarding the spatial orientation of the CS ostium, 14 (38%) patients showed drainage to the TAP. This prevailed in males (13/21 patients; 62%), while, among females, the CS ostium emptied toward the TAP in only one (6%) of 16 patients.

## Discussion

### Anatomical and radiological considerations of left ventricular lead positioning

The mediastinum is the central compartment of the thoracic cavity, consisting of hollow visceral structures filled with liquid or air and connected by soft connective tissue infiltrated by fat.^22^ The interpretation of radiological mediastinal shadows and details of cardiovascular anatomy facilitates the correct identification of the CS structures during transvenous LV lead implantation.

The CS is responsible for the venous drainage of the heart. It lies in and is the main constituent of the posterior portion of the atrioventricular groove. CS receives the venous drainage of the anterior half of the interventricular septum and the LV anterior wall through the great cardiac vein, which opens into the left extremity of the CS. The left atrium drains blood into CS via small atrial veins and the oblique vein of left atrium (the vein of Marshall). The lateral wall and a part of the LV posterior region drain blood via the left marginal vein.^13,22,23^ The rest of the LV posterior region and the posterior half of the interventricular septum drain blood mainly through the left posterior or the middle cardiac vein. At its final portion, the CS receives right venous drainage through the small cardiac vein.

**Figure 4:**
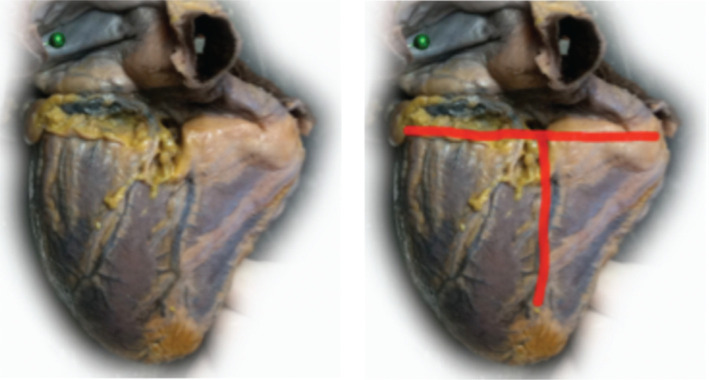
Diaphragmatic view of the normal heart. Observe the “T” shape formed by the CS (horizontal line) and the interventricular sulcus (vertical line). (This photograph was originally presented in the first author’s doctoral dissertation.)

**Figure 5:**
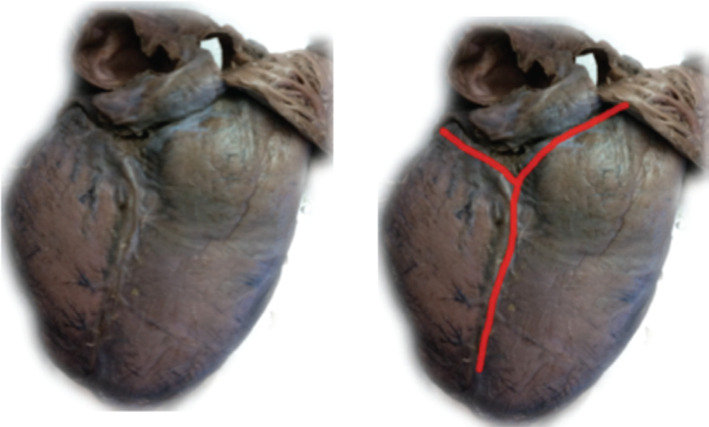
Diaphragmatic view of an enlarged heart. Observe the “Y” shape formed by the CS and the interventricular sulcus. (The photograph was originally presented in the first author’s doctoral dissertation.)

**Figure 6:**
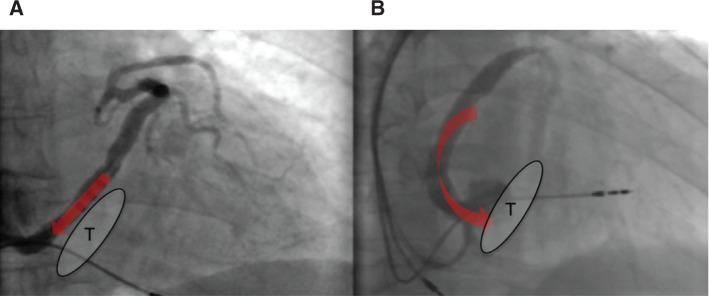
Two different CS phlebographies in the 30° RAO projection showing blood flow drainage. **A:** Blood flow points to the venous sinus. **B:** Blood flow points to the inlet of the right ventricle. T: tricuspid annular plane.

In normal hearts, the atrioventricular groove with CS and the ventricular septal plane typically form a “T” shape as shown in **Figure 4**. In some cases, a “Y” shape can be seen **(Figure 5)** because the CS is situated superior to the atrioventricular groove and is turned posterosuperior to the left atrium. Whether the “Y” shape may be a consequence of an enlarged left or right ventricle or is the result of embryonic development is unknown. With the “Y” shape, the CS ostium, commonly situated in the lower part of the right atrial septum, may change its spatial orientation, with important implications for procedures requiring CS catheterization such as CRT or electrophysiological study. In conventional orientation, CS directs the blood flow toward the venous sinus but, in cases of geometry distortion, it may direct the blood to the inlet of the right ventricle and the TAP as seen in **Figure 6**. The apex of the heart points approximately 45° to the sagittal and coronal planes (ie, 45° to the left and forward). An enlarged LV chamber tends to tilt the axis of the heart more medial and posteriorly and can thus change the orientation of the axis of the CS. The concomitant enlargement of the right ventricle tends to divert the left ventricle further back, which can additionally shift the axis of the CS. In the LAO projection, the septum adopts a more axial point, with the right cavities located rightmost and left cavities located leftmost of the observer **(Figure 3)**. In this way, the CS adopts a posterior upward trend related to the back of the patient, whereas the CS ostium is moved toward the observer. In the RAO view, the heart is projected from the side or transversally; therefore, the right chambers are available in the front, overlapping the left chambers. The CS ostium is again drawn nearer to the observer. Precisely in this projection, the geometric deformation imposed by dilated cardiomyopathy diverts the direction of the axis of the CS to the back (ie, toward the column and to the observer’s right). It is not uncommon that, in these conditions, the blood flows from the CS toward the TAP **(Figure 6)**.

**Figure 7:**
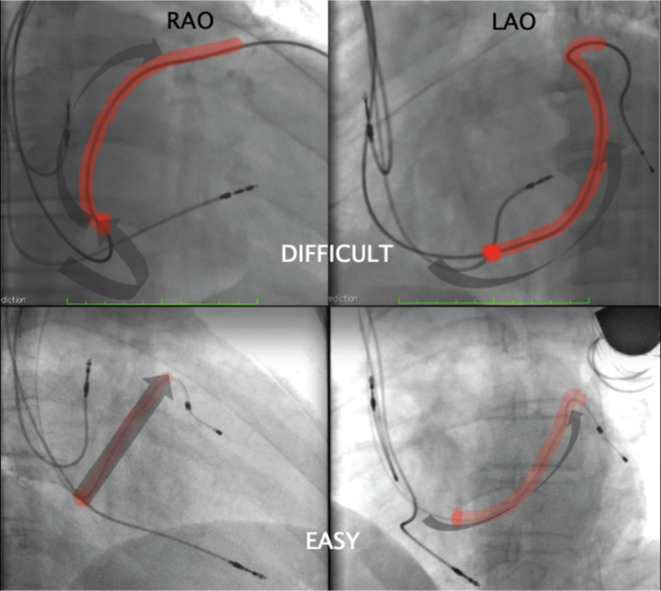
LV lead positioning view on radioscopy in two different patients (30° RAO and 30° LAO projections). The top is considered a difficult case and the bottom is considered an easy case with respect to CS catheterization and lead delivery.

In the population studied, there was a difference between the sexes in the spatial orientation of the CS ostium, with the direction to the TAP being more common in males and very uncommon in females. It is not possible to affirm that the differences observed here are solely related to anatomical variations between the sexes or the etiology of cardiomyopathies, since this was not a designed endpoint. We also observed that cases of CS ostium with TAP orientation initially presented as more difficult cases considering catheterization; this aspect was neutralized at the end of the study. Indeed, the time for LV lead positioning and the subjective perception of difficulty were higher for cases in which the CS ostium pointed to the TAP **(Table 4 and Figure 7)**.

### Accessing coronary sinus ostium

The main limiting factor for accessing the CS ostium is operator experience.^24^ Among several techniques, three are frequently used in medical practice. The first and the one most commonly used by electrophysiologists relies on the AP or 30° LAO view to “fish” the CS with a catheter or a preshaped lead. It is possible to form lead guides for this purpose or to use a special catheter. Some surgeons continuously record the intracavitary electrogram associated with radiological imaging to determine the CS position. The electrogram can be obtained from the LV lead (which can decrease procedure time) or with the aid of an electrophysiology catheter.^25^ The third methodology (phlebography) is standard for interventional cardiologists and consists of making small injections of contrast near the atrioventricular groove to visualize the CS ostium. This is generally done in AP or 30° LAO projections.

Commonly, CS can be accessed by trial and error. Not infrequently, the CS is even accessed unintentionally during conventional RV lead implantation. Currently, successful CS catheterization and LV lead placement seem to be more related to the anatomical aspects of the dilated heart than the type of resource used. Furthermore, the LV lead profile and its intimate relationship with the cardiac venous system can be decisive for procedure success.^24,26^

After reviewing a series of phlebographies in our cohort, we observed that difficult access to the CS was mainly attributable to the altered spatial orientation of the CS ostium (ie, if the CS ostium points to the inlet of the right ventricle) **(Figure 6)**. We moved to explore a radiolucent area in the 30° RAO projection located toward the column and upward following our indicative phlebography findings. A 30° RAO projection becomes a good key point for LV lead positioning in such a context.

Gonzalez-Vasserot et al. analyzed the CS drainage in a study of the venous phase of angiography in 35 patients.^27^ They used a 30° RAO view and the radiolucent area as described by Josephson.^21^ The authors suggested that the anatomical location of the CS in this projection may be used to facilitate interventional procedures that require access to the CS. However, the study included only four patients with dilated cardiomyopathy and the findings could not be extrapolated to patients with dilated hearts. In another study, Da Costa et al. used 64-slice computed tomography to analyze the cardiac anatomy of patients with difficult CRT implantation; the only anatomical factor predicting implantation difficulty was a greater distance of the CS ostium from the floor of the right atrium.^28^ Macias et al. reported LV lead implantation failure in 26 (12.3%) of 212 patients.^29^ During logistic regression analysis, the presence of permanent atrial fibrillation and a larger left atrium diameter in the AP view were independent predictors of a failed implant.

In our study, the CS ostium oriented toward the inlet of the right ventricle was associated with LV lead implantation difficulty. The 30° RAO projection, on top of the AP and 30° LAO projections, allowed for better assessment of the geometric distortion of the CS to facilitate its catheterization in a group of patients with dilated cardiomyopathy.

## Conclusion

This study is an exploratory study of a single-center experience and does not pretend to extrapolate its limitations. The existence of numerous techniques shows that there is no gold standard for transvenous LV lead placement in patients undergoing CRT implantation. Despite all the evolution that has occurred in CRT, the procedure remains dependent on fluoroscopy and the anatomical and radiological knowledge acquired in clinical practice. The methodology we discuss is based on the simple application of knowledge of radiological and anatomical aspects of the CS in dilated cardiomyopathy. AP, LAO, and RAO projections should always be standard on CRT. The RAO projection was, in this study, useful in the access of difficult CS ostia for LV lead positioning, especially when the CS ostium pointed to the TAP, particularly in males. During CRT procedures, the RAO projection may serve as a means to complete more time-consuming technically complex cases.

## Figures and Tables

**Table 1:** Patient Characteristics at Baseline, Procedure Success, and Time Intervals

Characteristic	Total (n = 38)	Male (n = 21)	Female (n = 17)	p-value
Age, years				0.243*
Mean ± SD (min–max)	61 ± 10 (42–81)	59 ± 11 (42–81)	63 ± 9 (47–78)	
Median (IQR)	61.5 (52–68)	60 (50–69)	63 (57–68)	
LVEF, %				0.008*
Mean ± SD (min–max)	31 ± 6 (17–38)	28 ± 6 (17–38)	34 ± 3 (25–38)	
Median (IQR)	32 (28–34)	30 (22–34)	34 (32–36)	
NYHA class, mean ± SD	3.4 ± 0.4	3.6 ± 0.4	3.2 ± 0.3	0.008*
Class III, n (%)	27 (71.1)	11 (52.4)	16 (94.1)	
Class IV, n (%)	11 (28.9)	10 (47.6)	1 (5.9)	
Etiology, n (%)				
Idiopathic	6 (15.8)	5 (23.8)	1 (5.9)	
Ischemic	11 (28.9)	7 (33.3)	4 (23.5)	
Chagasic	5 (13.2)	0	5 (29.4)	
Ischemic and chagasic	1 (2.6)	1 (4.8)	0	
Valvular	4 (10.5)	4 (19, 0)	0	
Hypertensive	9 (23.7)	3 (14.3)	6 (35.3)	
Myocarditis	1 (2.6)	1 (4.8)	0	
ARVD	1 (2.6)	0	1 (5.9)	
Implanted device, n (%)				
CRT	25 (65.8)	12 (57.1)	13 (76.5)	
CRT without atrial lead	3 (7.9)	3 (14.3)	0	
CRT-D	8 (21.1)	5 (23.8)	3 (17.6)	
CRT-D without atrial lead	2 (5.3)	1 (4.8)	1 (5.9)	
Procedure success rates, n (%)^†^				
Stable CS catheterization	37 (94.9)	21 (100.0)	16 (88.9)	0.387*
Final LV lead position	36 (92.3)	21 (100.0)	15 (83.3)	0.526*
Time interval, min, mean ± SD (min–max)				
From puncture to stable CS catheterization	17 ± 22 (2–98)	20 ± 28 (2–98)	14 ± 9 (4–30)	0.387*
For 37 successful catheterizations, median (IQR)	8 (5–23)	6 (4–26)	12 (6–21)	
From stable CS catheterization to final LV	30 ± 22 (10–107)	32 ± 26 (10–107)	26 ± 14 (10–62)	0.950*
Lead position (for 36 successful implants), median (IQR)	25 (17–31)	21 (19–5)	25 (14–31)	
From puncture to final LV lead position	47 ± 29 (13–121)	52 ± 35 (13–121)	40 ± 18 (16–76)	0.526*
For 36 successful implants, median (IQR)	40 (25–55)	37 (26–69)	42 (24–53)	

ARVD: arrhythmogenic right ventricular dysplasia; CS: coronary sinus; CRT-D: CRT with defibrillator backup; IQR: interquartile range; LV: left ventricular; LVEF: left ventricular ejection fraction; NYHA: New York Heart Association; SD: standard deviation.

*Mann–Whitney U test.

^†^A total of 39 implantation procedures were included in the analyses, as one woman underwent two procedures.

**Table 2:** Time Intervals as Function of LVEF (Median)

Time Interval, min	LVEF ≤ 31% (n = 14)	LVEF > 31% (n = 23)	p-value
From puncture to stable CS catheterization*			0.284***
Mean ± SD (min–max)	21 ± 24 (2–98)	14 ± 20 (3–97)	
Median (IQR)	19 (6–27)	7 (4–17)	
From puncture to final LV lead position**			0.860***
Mean ± SD (min–max)	48 ± 31 (13–121)	46 ± 28 (17–115)	
Median (IQR)	44 (28–57)	36 (25–57)	

CS: coronary sinus; IQR: interquartile range; LV: left ventricular; LVEF: left ventricular ejection fraction; SD: standard deviation.

*Excluding one patient with anomalous drainage of the CS into the left atrium and the redo procedure (total = 37 patients).

**Excluding one patient with anomalous drainage of the CS into the left atrium and the redo procedure and one patient with unpreventable phrenic nerve stimulation (total = 36 patients).

***Mann–Whitney U test.

**Table 3:** Time Intervals as Function of NYHA Class

Time Interval, min	NYHA III (n = 26)	NYHA IV (n = 11)	p-value
From puncture to stable CS catheterization*			0.612***
Mean ± SD (min–max)	16 ± 19 (3–98)	19 ± 27 (2–97)	
Median (IQR)	9 (6–23)	6 (4–23)	
From puncture to final LV lead position**			0.416***
Mean ± SD (min–max)	43 ± 24 (16–121)	57 ± 38 (13–115)	
Median (IQR)	36 (25–53)	45 (30–107)	

Abbreviations: CS: coronary sinus; IQR: interquartile range; LV: left ventricular; NYHA: New York Heart Association; SD: standard deviation.

*Excluding one patient with anomalous drainage of the CS into the left atrium and the redo procedure (total = 37 patients).

**Excluding one patient with anomalous drainage of the CS into the left atrium and the redo procedure and one patient with unpreventable phrenic nerve stimulation (total = 36 patients).

***Mann–Whitney U test.

**Table 4:** Time Intervals as Function of CS Ostium to the TAP

Time interval, min	No CS to the TAP (n = 22)	Yes CS to the TAP (n = 14)	p-value
From puncture to stable CS catheterization*			
Mean ± SD (min–max)	11 ± 9 (3–33)	27 ± 32 (2–98)	0.180**
Median (IQR)	8 (4–15)	21 (5–28)	
From puncture to final LV lead position*			
Mean ± SD (min–max)	35 ± 17 (13–76)	66 ± 34 (25–121)	0.002**
Median (IQR)	30 (22–49)	53 (37–108)	

CS: coronary sinus; IQR: interquartile range; SD: standard deviation; TAP: tricuspid annular plane.

*Excluding one patient with anomalous drainage of the CS into the left atrium and the redo procedure and one patient with unpreventable phrenic nerve stimulation (total = 36 patients).

**Mann–Whitney U test.

## References

[1] Cazeau S, Leclercq C, Lavergne T (2001). Effects of multisite biventricular pacing in patients with heart failure and intraventricular conduction delay.. N Engl J Med..

[2] Abraham WT, Fisher WG, Smith AL (2002). Cardiac resynchronization in chronic heart failure.. N Engl J Med..

[3] Cleland JG, Daubert JC, Erdmann E (2005). The effect of cardiac resynchronization on morbidity and mortality in heart failure.. N Engl J Med..

[4] Lindenfeld J, Feldman AM, Saxon L (2007). Effects of cardiac resynchronization therapy with or without a defibrillator on survival and hospitalizations in patients with New York Heart Association class IV heart failure.. Circulation..

[5] Auricchio A, Metra M, Gasparini M (2007). Long-term survival of patients with heart failure and ventricular conduction delay treated with cardiac resynchronization therapy.. Am J Cardiol..

[6] Linde C, Abraham WT, Gold MR, St John SM, Ghio S, Daubert C (2008). Randomized trial of cardiac resynchronization in mildly symptomatic heart failure patients and in asymptomatic patients with left ventricular dysfunction and previous heart failure symptoms.. J Am Coll Cardiol..

[7] Moss AJ, Hall WJ, Cannom DS (2009). Cardiac-resynchronization therapy for the prevention of heart-failure events.. N Engl J Med..

[8] Tang AS, Wells GA, Talajic M (2010). Cardiac-resynchronization therapy for mild-to-moderate heart failure.. N Engl J Med..

[9] Daubert JC, Saxon L, Adamson PB (2012). 2012 EHRA/HRS expert consensus statement on cardiac resynchronization therapy in heart failure: implant and follow-up recommendations and management.. Heart Rhythm..

[10] Vijayaraman P, Chung MK, Dandamudi G (2018). His bundle pacing.. J Am Coll Cardiol..

[11] Alonso C, Leclercq C, d’Allonnes FR (2001). Six year experience of transvenous left ventricular lead implantation for permanent biventricular pacing in patients with advanced heart failure: technical aspects.. Heart..

[12] El-Maasarany S, Ferrett CG, Firth A, Sheppard M, Henein MY (2005). The coronary sinus conduit function: anatomical study (relationship to adjacent structures).. Europace..

[13] Loukas M, Bilinsky S, Bilinsky E, el-Sedfy A, Anderson RH (2009). Cardiac veins: a review of the literature.. Clin Anat..

[14] Alonso C (2009). In the field of cardiac resynchronization therapy is left ventricular pacing via the coronary sinus a mature technique.. Europace..

[15] Daubert JC, Ritter P, Le BH (1998). Permanent left ventricular pacing with transvenous leads inserted into the coronary veins.. Pacing Clin Electrophysiol..

[16] Blanc JJ, Benditt DG, Gilard M, Etienne Y, Mansourati J, Lurie KG (1998). A method for permanent transvenous left ventricular pacing.. Pacing Clin Electrophysiol..

[17] Walker S, Levy T, Rex S, Paul VE (1999). The use of a “side-wire” permanent transvenous pacing electrode for left ventricular pacing.. Europace..

[18] Yu CM, Miu R (2002). A new technique for the transvenous implantation of the over-the-wire left ventricular pacing lead in a patient with heart failure.. J Interv Card Electrophysiol..

[19] Daoud EG, Kalbfleisch SJ, Hummel JD (2002). Implantation techniques and chronic lead parameters of biventricular pacing dual-chamber defibrillators.. J Cardiovasc Electrophysiol..

[20] Daubert JC, Behar N, Martins RP, Mabo P, Leclercq C (2017). Avoiding non-responders to cardiac resynchronization therapy: a practical guide.. Eur Heart J..

[21] Josephson ME (2001). Clinical Cardiac Electrophysiology: Techniques and Interpretations.

[22] Moore KL, Arthur FD (2008). Clinically Oriented Anatomy..

[23] von Lüdinghausen M, Schott C, Meerbaum S (1990). Microanatomy of the Human Coronary Sinus and its Major Tributaries..

[24] Lau EW (2009). Achieving permanent left ventricular pacing-options and choice.. Pacing Clin Electrophysiol..

[25] Souza FS, Mortati NL, Braile DM (2006). Technical aspects of coronary sinus catheterization based on the atrial component of the intracavitary electrogram and radiological anatomy during the implantation procedure of a biventricular pacemaker.. Arq Bras Cardiol..

[26] Zhao X, Burger M, Liu Y (2011). Simulation of LV pacemaker lead in marginal vein: potential risk factors for acute dislodgement.. J Biomech Eng..

[27] Gonzalez-Vasserot M, Gnoatto M, Merino JL (2008). The inferior radiolucent area within the cardiac silhouette: validation as a landmark for coronary sinus catheterization.. Pacing Clin Electrophysiol..

[28] Da Costa A, Gate-Martinet A, Rouffiange P (2012). Anatomical factors involved in difficult cardiac resynchronization therapy procedure: a non-invasive study using dual-source 64-multi-slice computed tomography.. Europace..

[29] Macias A, Garcia-Bolao I, Diaz-Infante E (2007). Cardiac resynchronization therapy: predictive factors of unsuccessful left ventricular lead implant.. Eur Heart J..

